# Diagnosis of head-and-neck cancer from exhaled breath

**DOI:** 10.1038/bjc.2011.128

**Published:** 2011-04-19

**Authors:** M Hakim, S Billan, U Tisch, G Peng, I Dvrokind, O Marom, R Abdah-Bortnyak, A Kuten, H Haick

**Affiliations:** 1 The Department of Chemical Engineering and Russell Berrie Nanotechnology Institute, Technion – Israel Institute of Technology, Haifa 32000, Israel; 2 Oncology Division, Rambam Health Care Campus, Haifa 31096, Israel; 3 Bruce Rappaport Faculty of Medicine, Technion – Israel Institute of Technology, Haifa 31096, Israel

**Keywords:** head-and-neck cancer, lung cancer, volatile biomarker, breath, sensor, GC–MS

## Abstract

**Background:**

Head-and-neck cancer (HNC) is the eighth most common malignancy worldwide. It is often diagnosed late due to a lack of screening methods and overall cure is achieved in <50% of patients. Head-and-neck cancer sufferers often develop a second primary tumour that can affect the entire aero-digestive tract, mostly HNC or lung cancer (LC), making lifelong follow-up necessary.

**Methods:**

Alveolar breath was collected from 87 volunteers (HNC and LC patients and healthy controls) in a cross-sectional clinical trial. The discriminative power of a tailor-made Nanoscale Artificial Nose (NA-NOSE) based on an array of five gold nanoparticle sensors was tested, using 62 breath samples. The NA-NOSE signals were analysed to detect statistically significant differences between the sub-populations using (i) principal component analysis with ANOVA and Student's *t*-test and (ii) support vector machines and cross-validation. The identification of NA-NOSE patterns was supported by comparative analysis of the chemical composition of the breath through gas chromatography in conjunction with mass spectrometry (GC–MS), using 40 breath samples.

**Results:**

The NA-NOSE could clearly distinguish between (i) HNC patients and healthy controls, (ii) LC patients and healthy controls, and (iii) HNC and LC patients. The GC–MS analysis showed statistically significant differences in the chemical composition of the breath of the three groups.

**Conclusion:**

The presented results could lead to the development of a cost-effective, fast, and reliable method for the differential diagnosis of HNC that is based on breath testing with an NA-NOSE, with a future potential as screening tool.

Head-and-neck cancer (HNC) is the eighth most common cancer worldwide and a major cause of cancer mortality (Parkin *et al*, 2005; Pai and Westra, 2009). It comprises a group of diverse tumour types arising from various anatomic structures including the craniofacial bones, soft tissues, salivary glands, skin, and mucosal membranes (Pai and Westra, 2009). More than 90% of all HNCs are squamous cell carcinoma that arise from mucosa lining the oral cavity, oropharynx, hypopharynx, larynx, sinonasal tract, and nasophaynx (Pai and Westra, 2009; Marur *et al*, 2010).

Head-and-neck cancer is a particularly distressing human cancer, because both disease and treatment profoundly interfere with everyday functioning such as eating, breathing, and speech, and may lead to severe disfigurement (Hanna and Sherman, 1999). The diagnosis of HNC is not trivial and requires specialist settings. A general medical evaluation has to be performed, including a thorough head-and-neck examination by one or more physicians, followed by contrast-enhanced computed tomography (CT) and/or magnetic resonance imaging (MRI), and biopsies (Mendenhall *et al*, 2008). Head-and-neck cancer lacks specific symptoms and has a large number of clinical phenotypes so that patients often turn to a general practitioner or a dentist first, instead of a HNC specialist. As a result, diagnosis may be delayed. Two thirds of the patients are diagnosed with locally advanced or metastatic disease (stages III and IV) (Nagaraj, 2009). Long-term survival rates for advanced HNC are low and have not improved significantly over the last decades (Schweitzer *et al*, 2010). Overall cure is achieved in <50% of all patients, despite recent advances in surgery and radiotherapy (Goerner *et al*, 2010). In addition, HNC patients often develop a second primary cancer with an annual rate of 3–7% that is usually located again in the head-and-neck area or in the lung, making lifelong follow-up of HNC survivors necessary (Ridge *et al*, 2010). Therefore, reliable point-of-care diagnostic tests for HNC are urgently needed to improve survival, to reduce the need of mutilate surgery and, hence, to minimise the impact of HNC and HNC treatment on everyday functioning.

The vast majority of HNC cases appear to be induced by chronic exposure to carcinogens enclosed in all forms of tobacco, synergised by alcohol consumptions and/or associated with oncogenic human papillomaviruses (Schweitzer *et al*, 2010). The exposure to the carcinogens causes progressive accumulation of genetic and epigenetic changes in the squamous cells of the head or neck (Mendenhall *et al*, 2008; Schmutzhard *et al*, 2008; Nagaraj, 2009; Goerner *et al*, 2010), leading to cellular oxidative stress (Okunieff *et al*, 2005), and, thus, to the emission of cancer-specific volatile organic compounds (VOCs) into the blood (Schmutzhard *et al*, 2008). A part of the VOC biomarkers in the blood are likely to be transmitted to the alveolar exhaled breath through exchange via the lung. This possibility to detect cancer via VOC biomarkers in the exhaled breath is supported by several recent gas chromatography/mass spectroscopy (GC–MS) studies that have shown specific patterns of exhaled volatile biomarkers in lung cancer (LC) (Gordon *et al*, 1985; Preti *et al*, 1988; Phillips *et al*, 1999, 2003; Peng *et al*, 2009, 2010; Fuchs *et al*, 2010), breast cancer (Phillips *et al*, 2003, 2006; Peng *et al*, 2010; Shuster *et al*, 2011), prostate cancer (Peng *et al*, 2010), and colorectal cancer (Peng *et al*, 2010). These are mostly C_4_–C_20_ straight and mono-methylated alkanes, in addition to certain benzene derivatives (Gordon *et al*, 1985; Preti *et al*, 1988). It has also been shown that this information can be used to classify subjects as cancerous or not (Phillips *et al*, 1999, 2003, 2006; Peng *et al*, 2009, 2010). The compounds of interest are generally found in healthy human breath, but can be seen in distinctively different mixture compositions in the breath of cancer patients (Wang, 2005, personal communication). Schmutzhard *et al* have recently shown evidence that concentration profiles of volatile biomarkers in exhaled breath may also be used to distinguish HNC patients from both high-risk (i.e., heavy smokers and drinkers) and low-risk healthy controls. They measured the concentration of the HNC-specific volatile biomarkers, mainly hydrocarbons, in particular alkanes, alkenes, alcohols, ketones, organic acids, by proton transfer reaction-mass spectrometry (Schmutzhard *et al*, 2008). However, the compounds were not identified by name.

Haick and co-workers have recently designed a Nanoscale Artificial NOSE (NA-NOSE; see Materials and Methods) that distinguished between the breath of breast, lung, colon, and prostate cancer patients and healthy controls, irrespective of the patients' age, gender, lifestyle, smoking habits, and other confounding factors (Peng *et al*, 2009, 2010; Tisch and Haick, 2010). Here, we show that a tailor-made NA-NOSE can be used to distinguish between HNC and healthy controls, LC and healthy controls, and HNC and LC. A small-scale clinical trial is presented as proof-of-concept. The presented results could form the basis of a future point-of-care diagnostic test for a comprehensive HNC management, including differentiated diagnosis to enable optimal treatment at minimal interference with basic functions, and may hold future potential as screening test for at-risk populations and lifelong follow-up of HNC survivors.

## Materials and methods

### Human subjects

The test population included 87 volunteers (22 HNC patients, 25 LC patients, and 40 healthy controls), aged 24–78 years, which had given written informed consent according to the guidelines of the Rambam Healthcare Campus and Technion's committee for supervision of human experiments, Haifa, Israel. One fourth of the tested HNC patients had early-stage (stages I and II) disease. The clinical characteristics of the test population are summarised in Table 1. The complete clinical details of each volunteer can be found in the Supporting Online Information (SOI), Supplementary Table S1. All cancer patients were recruited from the Oncology Division, Rambam Health Care Campus (Haifa, Israel), after conventional diagnosis followed by biopsy and before any treatment. Most of the healthy controls were recruited from the same clinical environment.

### Study design

The clinical study was cross-sectional. Twenty-two patients with proven diagnosis of HNC were referred to the Oncology Division at Rambam Health Care Campus (see Table 1). Diagnosis was achieved through physical examination in combination with CT and/or MRI, followed by biopsy. The conventional diagnosis was used as a reference standard. All the breath samples were collected before any treatment.

The HNC patients were compared with two control groups: (i) 40 healthy subjects and (ii) 25 LC patients. The recruitment of the LC control group was described in Peng *et al* (2010). Note that the LC control group was age- and gender-matched to the HNC study group, but the healthy control group was not. This relaxation of the control-group criteria is acceptable, as the NA-NOSE sensors have been specially tailored to show little sensitivity to important confounding factors such as age, gender, and smoking habits (*cf.* sections Breath analysis using the NA-NOSE and Identification of HNC using the NA-NOSE; Supplementary Figures S1 and S2, SOI). No patient exclusion criteria were applied after recruitment.

### Breath collection

Exhaled alveolar breath was collected in a controlled manner from individuals with HNC, LC and from healthy subjects in the same room/atmosphere (see Supplementary Figure S3, SOI). The inhaled air was cleared of ambient contaminants by repeatedly inhaling to total lung capacity for 3–5 min through a mouthpiece (purchased from Eco Medics, Duerten, Switzerland) that contains a filter cartridge on the inspiratory port, thus greatly reducing the concentration of exogenous VOCs and removing 99.99% of the exogenous compounds from the air during inspiration. Since some typical hospital contaminations might be present in very large concentration, we sampled the unfiltered hospital air and disregarded the identified contaminants in our subsequent analysis. Immediately after the lung washout, subjects exhaled through a separate exhalation port of the mouthpiece against 10–15 cm H_2_O pressure to ensure closure of the vellum so that nasal entrainment of gas is excluded. Complementary experiments optimising the breath collection procedure have shown that the sampling methodology simply measures alveolar breath uncontaminated by upper airways release and exogenous compounds. Exhaled breath is a mixture of alveolar air and respiratory dead space air. The dead space was automatically filled into a separate bag and the alveolar breath into a 750-ml Mylar sampling bag (purchased from Eco Medics). It should be emphasised that the described breath collection is a single-step process that does not require the volunteer to take care of changing between the dead space and alveolar breath bags. The Mylar bags were re-used and thoroughly cleaned before each use with flowing N_2_ (99.999% purity) gas for 5–8 min. Notably, GC–MS in conjugation with solid-phase micro-extraction (SPME) has shown that this purification process eliminates 98% of contaminants and/or volatile biomarkers from the previous sample tested in a specific Mylar bag. In all, 1–2 bags were collected per test person for analysis with GC–MS and/or for analysis with the NA-NOSE – see below. All bags were analysed within 3 days from the time of breath collection (Peng *et al*, 2008, 2009, 2010). Note that currently the study of cancer biomarkers in exhaled breath suffers from a lack of standardisation of the breath collection and analysis. Amann *et al* (2010) have recently proposed a standardisation of the breath collection process that might be generally accepted in the future.

### Breath analysis using the NA-NOSE

Sixty-two breath samples were tested with an NA-NOSE (Peng *et al*, 2009, 2010; Tisch and Haick, 2010) developed by Haick and co-workers. The NA-NOSE is an artificial olfactory system based on an array of highly cross-reactive gas sensors that can identify and separate different odours, even if the odorants are present at very low concentrations and their differences are very subtle (see Supplementary Figure S2, SOI). Note, that the NA-NOSE sensors have been designed to show very little sensitivity to volatile biomarkers stemming from confounding factors such as age, gender, medication, smoking habits, and environmental effects, including long-time exposure to clinical environment (see Supplementary Figure S1, SOI, which was reproduced from Peng *et al* (2010)). This allowed us to relax the criteria for the healthy control population in terms of gender and age. The array contained five sensors that were based on spherical gold nanoparticles (GNPs) with tert-dodecanethiol, hexanethiol, 2-mercaptobenzoazole, 1-butanethiol, and 3-methyl-1-butanethiol ligands. These five sensors showed no or little overlap in average sensing signal to the breath samples from the three test groups, under consideration of the 95% confidence interval (CI) defined by 1.96 × s.e.. Each sensor showed a characteristic response to all (or to a certain subset) of the volatile biomarkers found in the exhaled breath samples. The sensing principle of the NA-NOSE was described in detail in Peng *et al* (2009, 2010). Note, however, that a different set of sensors was used in the present study.

### Breath analysis using GC–MS

The VOCs in the collected breath were identified through GC–MS analysis (GC-6890N; MS-5975; Agilent Technologies Ltd, Santa Clara, CA, USA) of 40 breath samples. Typical contaminants of the hospital environment were identified from the collected samples of unfiltered hospital air. The GC–MS analysis was preceded by SPME for pre-concentrating the volatile biomarkers in breath samples. A manual SPME holder with an extraction fibre was inserted into the Mylar bag for 30 min before being delivered to the GC–MS. Fibres with polydimethylsiloxane-divinylbenzene coating obtained from Sigma-Aldrich (Rehovot, Israel). The extracted fibre in the manual SPME holder was inserted into the injector of the GC (splitless mode). The following oven profile was used: 60°C, 2 min, 8°C per min to 100°C, 15°C per min to 120°C, 8°C per min to 180°C, 15°C per min to 200°C, and 8°C per min to 225°C. A capillary column H5-5MS 5% phenyl methyl siloxane (30 m length, 0.25 mm i.d., 0.25 mm thickness from Agilent Technologies Ltd) was used. The column pressure was set at 8.22 PSI and the initial flow rate was 1.0 ml per min. Tentative identification of the VOCs was performed through spectral library match.

### Statistical analysis

#### NA-NOSE

The NA-NOSE signals were analysed with standard principal component analysis (PCA) (Roeck *et al*, 2008), which shows the variability of the experimental data and allows the distinction of tentative clusters through visual perception of the 2D principal component (PC) plots. Principal component analysis determines the linear combinations of the input values such that the maximum variance between all data points can be obtained in mutually orthogonal dimensions. Thus, PCA effectively reduces the multi-dimensional experimental data to its main components and, thus, improves the human perception of the data. However, PCA does not classify the data. Objective classification was achieved by studying the statistical distribution of the first two principal components (PC1 and PC2) using two different approaches. In the first approach, one-way ANOVA of PC1 was conducted to compare principal component scores among the different breath patterns. Separation between the test groups was analysed using the Student's *t*-test. In a complimentary approach, support vector machines (SVMs) was used to classify the principal component data and cross-validation was utilised to evaluate the specificity and sensitivity (Cortes and Vapnik, 1995; Hall *et al*, 2009). The SVM analysis is a supervised learning method that finds the best separating line between two data sets. It can be used for data classification and pattern recognition (Cortes and Vapnik, 1995). The advantage of SVM over ANOVA combined with Student's *t*-test is that it can be applied to multi-dimensional data (here PC1 and PC2), and that it does not require normal distribution of the data points around the average value. Therefore, SVM is more suitable when dealing with smaller data sets. The three binary data sets (HNC and healthy states, LC and healthy states, and HNC and LC states) were analysed by building a multi-class classifier based on a linear nu-SVC SVM classifier on PC1 and PC2 (Hall *et al*, 2009). Cross-validation was utilised to evaluate the specificity and sensitivity by randomly dividing the samples into two sets, which are then used as training and test set. All possible combinations of division into two sets are tested and the results are averaged. Because of the limited number of samples, we opted for a high number of folds: 30 out of 36 samples for distinguishing HNC and healthy states, 40 out of 46 for distinguishing LC and healthy states, and 40 out of 42 for distinguishing HNC and LC states. Note that the results were stable against changing the number of folds in the cross-validation.

#### GC–MS

The volatile biomarkers common for <80% of the healthy and/or cancer samples, as well as their average abundance with s.e., were identified by their masses and retention times, using the Automated Mass Spectral Deconvolution and Identification System (ADMIS) software. Standard principal component analysis was applied to the set of tentatived volatile HNC biomarkers (Roeck *et al*, 2008) to determine tentative patterns of HNC states. The volatile biomarkers that (i) were present in both <80% of the HNC and <80% of the healthy controls and (ii) that showed no overlap in average abundance (under consideration of the 95% CI=1.96 × s.e.) were used as input values for the PCA. The first two principal components were plotted and tentative clusters corresponding to the different test groups were identified through visual perception.

## Results and discussion

### Identification of HNC using the NA-NOSE

**Figure 1:**
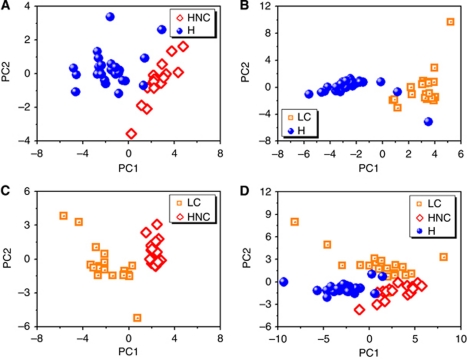
PCA plots of the PC1 and PC2 values of the five sensor NA-NOSE responses of (**A**) HNC and healthy sub-populations, (**B**) LC and healthy sub-populations, (**C**) HNC and LC, and (**D**) all patients: HNC, LC, and healthy controls. Each patient is represented by one point in plot. The first two principal components depicted contained 80, 67 and 70 and 66% for (**A**–**D**), respectively, of the total variance in the data. All test persons including the misclassified were considered in the statistical analysis.

Figure 1A shows the first two principal components that contain >80% of the variability within the data. A very clear separation between 16 tested HNC patients and 26 healthy subjects can be observed, with no overlap between the clusters for the small study population. As can be seen, the separation is almost entirely along the PC1 axis, with negative values for the healthy and positive values for the HNC states, respectively.

Note that PCA alone does not classify the data. The statistical distribution of the PC1 values was studied using ANOVA and Student's *t*-test. (Note of caution: the Student's *t*-test is based on normal distribution of the data points and equal variances within the two groups that are compared.) The PC1 values of the healthy and HNC test groups were distributed around −1.59 and 2.59, respectively (see Figure 1A and Table 2A). The s.d. (containing 68% of the PC1 values under the assumption of normal distribution) and the 99.9% CIs of the PC1 mean values are also listed in Table 2A. The CIs are relatively large, as a result of the small test population. Nevertheless, they do not overlap, but are, on the contrary, very well separated (*P*<0.0001; see Table 2A). Figure 1B shows that excellent discrimination is achieved also between LC and healthy states, using the same 5-sensor NA-NOSE. This is in agreement with our earlier results (Peng *et al*, 2010). The separation occurs almost entirely along the PC1 axis, with negative PC1 values for the healthy states and positive values (see Figure 1B and Table 2B) for the LC states, and with two healthy misclassified as LC. As before, very good separation was achieved even between the 99.9% CIs (*P*<0.0001; see Table 2B). Figure 1C shows that the NA-NOSE also achieved excellent separation between HNC and LC states along PC1, using the same NA-NOSE based on five GNP sensors, in contrast to the chemical analysis of the constituent compounds. The 99.9% CIs of the PC1 values for the two study groups were fully separated (*P*<0.0001; *cf.* Table 2C). These three tests are sufficient for the unambiguous identification of HNC, and the separation of HNC and LC states in a future screening breath test that might serve the same high-risk group (for HNC and LC) of tobacco users. Figure 1D shows that even HNC, LC, and healthy states together might be separated in the same statistical analysis, since they form three well-defined clusters in 2D principal component space. However, in this case PC1 and PC2 have to be considered for a full separation of the three clusters (*cf.* Table 1). Note, that PCA on only two of the three study groups (Figures 1A–C) is different from PCA on all three study groups (Figure 1D).

In a complimentary approach, we have used SVM analysis to find the best separating line between two data sets (Cortes and Vapnik, 1995). Support vector machine does not require normal distribution of the data points. We analysed the three binary data sets (HNC and healthy states, LC and healthy states, and HNC and LC states). The specificity and sensitivity were determined through cross-validation as described in the section Statistical analysis. The numbers of correct and incorrect patient classifications are listed in Tables 3A–C. For example, HNC classified as HNC are true positive (TP), HNC classified as healthy are false negative (FN), healthy classified as healthy are true negative (TN), and healthy classified as HNC are false positive (FP). From this, we can extract sensitivities of 100% and specificities of 92% for detecting both HNC and LC. The distinction between HNC and LC was even clearer, with sensitivity and specificity of 100%. However, the very encouraging results of this proof-of-concept study have to be verified in a larger clinical trial.

Table 1 shows the HNC, LC, and healthy study groups for the NA-NOSE analysis contain both smokers and non-smokers. However, while the HNC and LC groups contain more smokers, the number of non-smokers is higher in the healthy control group (see Table 1). As tobacco smoking could affect the chemical composition of the exhaled breath, we carefully excluded possible confounding effects of the smoking habits of the tested subjects on the presented results. For this purpose, a separate PCA analysis of the collective NA-NOSE response to the breath samples of the healthy control group (containing 7 smokers and 19 non-smokers) was performed. We observed, that the same NA-NOSE based on five GNP sensors that clearly identified HNC states (*cf*. Figure 1), showed no separation at all between smokers and non-smokers (see Supplementary Figure S2, SOI).

In this small-scale clinical trial, we have not attempted to distinguish between early-stage and late-stage HNC. Note, however, that a fifth of the tested and correctly classified HNC patients had early-stage disease (see Table 1 and Supplementary Table S1 in the SOI). This indicates that the NA-NOSE can identify HNC at an early stage (stages I and II) and opens exciting future prospects for application as a novel screening method.

### Identification of tentative volatile HNC biomarkers in the breath

**Figure 2:**
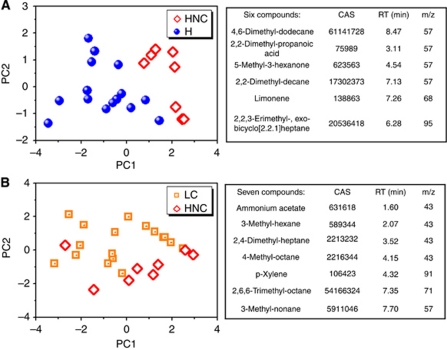
PCA of the PC1 and PC2 values resulting from statistical analysis of the abundance of volatile biomarkers determined by GC–MS/SPME analysis, using (**A**) six common volatile biomarkers for distinguishing HNC from healthy states; (**B**) seven common volatile biomarkers to distinguish HNC from LC. The compound names, masses, and CAS registry numbers are listed in the tables on the right of the PC plots.

GC–MS analysis was carried out as supportive method to validate the patterns that stem from response of the NA-NOSE to the breath samples of the HNC and LC patients, and healthy controls. The chemical analysis of the collected exhaled alveolar breath identified several substances that differ in average abundance in breath samples taken from the HNC patients and healthy controls. Hence, it is likely to consider these substances as potential volatile biomarkers of HNC. For this purpose, a representative subset of the collected breath samples were analysed (8 HNC, 15 healthy controls; see Table 1) using GC–MS in combination with SPME. The right panel of Figure 2A lists six common compounds that (i) are present in both >80% of HNC and >80% of healthy subjects and (ii) differ sufficiently in their average abundances in the two sub-populations (no overlap of the 95% CIs represented by 1.96 × s.e.,). Note that the identification of the biomarkers through spectral library match and retention times is tentative, because confirmation of identity through GC–MS analysis of reference substances was carried out only for p-xylene. However, the comparison between patients was based on compound masses and retention times.

Figure 2A shows that this combination of compounds allows the establishment of tentative HNC volatile fingerprints in principal component space. Very good separation can be observed between the clusters that are associated with HNC and healthy states. Note that hospital contaminants were identified from unfiltered air in the room where the sampling took place. The following typical hospital contaminants were found: 2-methyl-2-propanol, ethanol, and methyl-isobutyl-ketone. These substances were found in low abundance in <30% of the study population, indicating that the air filtration was efficient. Therefore, these substances were not considered in the analysis. Note that limonene and p-xylene have been mentioned as possible environmental contaminants in the literature (Amann *et al*, 2010), but were not detected in the sampled hospital air. Therefore, they were listed as potential biomarkers in Figure 2. Straight chain hydrocarbons are known to be products of lipid peroxidation (Miekisch *et al*, 2004). Lipid peroxidation is a chain reaction that is initiated by the removal of an allelic hydrogen atom through reactive oxygen species. The alkyl radicals undergo further reactions that generate saturated hydrocarbons. However, the metabolic origin of the detected compounds was not clarified in this study, and the proposed set of tentative HNC biomarkers should be handled with care.

We have previously reported that LC and healthy states can be distinguished using the concentration profiles of six tentative volatile breath biomarkers (for details, see Peng *et al* (2010)). What remains to be shown is that we can identify suitable compounds in the breath that would allow the distinction of HNC and LC, as HNC patients often develop a second primary lung tumour. We could not find compounds that (i) are present in >80% of both HNC and LC samples and (ii) do not overlap in abundance. However, we were able to identify a tentative set of seven compounds that are present in at least 80% of either HNC or LC states, and do not overlap in abundance (see the list in the right panel of Figure 2B). They allow moderate separation between HNC and LC, with one HNC state clearly misclassified as LC and a slight overlap at the separating line between the clusters.

It is important to verify that the proposed sets of tentative biomarkers are not related to smoking, because the HNC, LC, and healthy study groups for the GC–MS analysis are not well matched with regard to their smoking habits (see Table 1). The effect of smoking on the chemistry of exhaled breath has been studied by several groups; and a variety of breath VOCs has been associated with smoking (e.g., see Amann *et al* (2010), Fuchs *et al* (2010), Kischkel *et al* (2010), and references therein). In a separate pilot study, we have investigated smoking-related compounds in exhaled breath, using eight healthy smokers and eight healthy non-smoking controls that have never smoked in their lives (for details refer to Supplementary Table S2, SOI). Our preliminary results showed that the proposed tentative HNC biomarkers were not present in elevated abundance in the smoker test group. Differences to the smoking-related compounds in exhaled breath that were reported by other groups may be due to the different methods of pre-concentration, *viz.* the use of a different solid phase during SPME (Amann *et al*, 2010; Fuchs *et al*, 2010; Kischkel *et al*, 2010). However, the small sample size of the pilot study does not allow to confirm or to exclude any smoking biomarker as such. This separate study is ongoing and the results for an extended study population will be published elsewhere. Since this is so, we have carefully excluded possible confounding effects of the smoking habits of the tested subjects on the presented GC–MS results. For this purpose, we calculated three additional PCA maps, using the three proposed marker sets, for the subgroup of the LC patients, as a representative example. The LC group was selected, because it contained a sufficient number of well-distributed subjects (10 smokers and 7 non-smokers), while all the healthy controls studied by GC–MS were non-smokers and the HNC group were mostly smokers. Our PCA analysis of the LC study group confirmed that the three sets of VOCs that distinguished between (i) HNC and healthy states, (ii) HNC and LC states, and (iii) LC and healthy states, showed no separation at all between smokers and non-smokers (see Supplementary Figures S4 A–C, SOI).

The above GC–MS analysis has shown that the abundance of certain substances differs for the three study groups, that is, that there are certain differences in the average chemical composition of the breath, that may be detected with the NA-NOSE (see section Identification of HNC using an NA-NOSE). Note, however, that this study did not clarify whether the VOCs identified by GC–MS are indeed the same VOCs that cause the HNC-specific NA-NOSE patterns.

### Comparison between the discriminative power of the NA-NOSE and the chemical analysis through GC–MS

The superior discriminative abilities of the NA-NOSE, as compared with statistical analysis of the averaged abundance of the constituent compounds of the breath samples, detected by GC–MS/SPME, are in agreement with our earlier study (Peng *et al*, 2010), and can be understood if one considers the fundamental differences between the two methods. The NA-NOSE sensors are broadly cross-reactive and all respond (mainly) to the compounds of interest. While the responses to the same compound at a certain concentration are individually different between the constituent sensors, due to the chemical diversity of the GNP ligands, the signals to the mixture compounds that are present in the breath sample are additive, so that the overall signal of one sensor stems from a total tens to hundreds of ppb of cancer volatile biomarkers. Hence, the sensors' responses are less affected by noise than the detected (sub) ppb concentrations of the separate compounds in the GC–MS/SPME analysis. Also, the NA-NOSE sensors have been tuned, through suitable choice of the GNP ligands, to show very little sensitivity to volatile biomarkers that stem from confounding factors such as age, gender, medication, smoking habits, and other lifestyle factors (*cf.* Supplementary Figures S1 and S2, SOI; and Peng *et al* (2010)). This is particularly relevant to this study, and allowed us to relax the criteria for the healthy control population in terms of gender and age. In contrast, GC–MS detects also these confounding volatile biomarkers, which introduces noise into the measurement of the abundance of the compounds of interest and, hence, affects the overall accuracy of the method.

## Summary and conclusion

We have delivered a proof-of-concept for the ability of a tailor-made NA-NOSE to identify unambiguously HNC patients in a population that contains healthy subjects and LC patients. In a complimentary approach, we have identified two tentative sets of six and seven VOCs that allowed distinguishing HNC from healthy states and LC states, respectively, using GC–MS/SPME. An extended, double-blind study with a larger study population is necessary to fully validate the method. The NA-NOSE proved superior to GC–MS in separating HNC and healthy, LC and healthy, and HNC and LC states. This method could form the basis of a future cost-effective, fast, and reliable point-of-care diagnostic test for HNC that could be available to general practitioners or dentists, outside of specialist head-and-neck clinics. Moreover, this method holds potential as screening test for populations at risk of developing either HNC or LC, and as follow-up medical test for HNC survivors who tend to develop a second primary cancer. Considering that currently no adequate diagnostic and screening tests for HNC are available, this approach could have a significant impact on HNC mortality in the future.

## Figures and Tables

**Table 1:** Summary of the clinical characteristics of 87 volunteers that were tested for this study

		**NA-NOSE**			**GC–MS**								
	**No. of patients**	**Total**	**Smokers**	**Non- smokers**	**Total**	**Smokers**	**Non- smokers**	**Average age±s.d.**	**M:F ratio**	**Stage I**	**Stage II**	**Stage III**	**Stage IV**
HNC	22	16	10	6	8	6	2	60±9	19:3	3	1	3	15
LC	25	20	12	8	17	10	7	66±8	22:3	—	—	12	12
Healthy	40	26	7	19	15	0	15	45±13	17:23				

Abbreviations: NA-NOSE=Nanoscale Artificial Nose; GC–MS=gas chromatography/mass spectroscopy; HNC=head-and-neck cancer; LC=lung cancer; M:F=male:female.

For the complete clinical details of all volunteers, please refer to SOI, Supplementary Table S1.

**Table 2:** One-way ANOVA analysis of the PC1 values for the correctly classified subjects and Student's *t*-test for detecting statistically significant differences

**Sub- population**	**No. of subjects**	**Mean PC1**	**s.d.**	**s.e.**	**Lower 99.9% CL**	**Upper 99.9% CL**	**Difference of the PC1 mean values**	**Lower 99.9% CL difference**	**Upper 99.9% CL difference**	***P*-value**
(*A)*										
HNC	16	2.59	1.17	0.29	1.20	4.00	4.20	2.58	5.81	<0.0001
Healthy	26	−1.59	1.77	0.35	−2.70	−0.50				
										
*(B)*										
LC	20	3.13	1.13	0.25	2.15	4.11	5.53	4.00	7.08	<0.0001
Healthy	26	−2.41	1.82	0.36	−3.74	−1.08				
										
*(C)*										
HNC	16	2.20	0.34	0.08	1.86	2.55	3.97	5.45	2.48	<0.0001
LC	20	−1.76	1.69	0.38	−3.23	−0.29				
										
*(D)*										
HNC	16	2.92	1.93	0.48	0.96	4.89				
LC	20	1.25	3.48	0.78	−1.77	4.28				
Healthy	26	−2.76	2.13	0.42	−4.32	−1.21				

Abbreviations: ANOVA=analysis of variance; PC1=first principal component.

Mean value of PC1, s.d., as well as upper and lower 99.9% confidence limit (CL), differences in PC1 values and CLs, and *P*-values for (A) healthy controls and head-and-neck cancer (HNC) patients, (B) healthy controls and lung cancer (LC) patients, (C) HNC and LC patients, and (D) healthy controls, LC and HNC patients.

**Table 3:** Number of correct and incorrect patient classifications using supportive vector machine (SVM) and cross validation

**(A)**	**Classified as healthy**	**Classified as HNC**
Healthy	24	2
HNC	0	16
		
**(B)**	**Classified as healthy**	**Classified as LC**
Healthy	24	2
LC	0	20
		
**(C)**	**Classified as HNC**	**Classified as LC**
HNC	16	0
LC	0	20

Abbreviations: HNC=head-and-neck cancer; LC=lung cancer.

The accuracy of the diagnostic method was (A) 95% (B) 96% and (C) 100%.
