# Supplementary material for: Diagnosis of head-and-neck cancer from exhaled breath
Source: Br J Cancer. 2011 Apr 19;104(10):1649–55. doi: 10.1038/bjc.2011.128 (PMC3101906; doi:10.1038/bjc.2011.128)
Supplement: Supplementary Information [file bjc2011128x1.doc]

*Supporting Online Information for*

**DIAGNOSIS OF HEAD-AND-NECK CANCER FROM EXHALED BREATH**

Meggie Hakim^1^, Salem Billan^2^, Ulrike Tisch^1*^, Gang Peng^1^, Irena Dvrokind^1^, Ophir Marom^1^, Roxolyana Abdah-Bortnyak^3^, Abraham Kuten,^2,3^ and Hossam Haick^1*^

^1^ The Department of Chemical Engineering and Russell Berrie Nanotechnology Institute, Technion – Israel Institute of Technology, Haifa 32000, Israel.

*^2^ Oncology Division, Rambam Health Care Campus, Haifa 31096, Israel.*

*^3^ Bruce Rappaport Faculty of Medicine, Technion – Israel Institute of Technology, Haifa 31096, Israel.*

***Correspondence to:** Prof. Hossam Haick and Dr. Ulrike Tisch, the Department of Chemical Engineering and Russell Berrie Nanotechnology Institute, Technion – Israel Institute of technology, Haifa 32000, Israel. Tel: +972 (4) 8293087; Fax: +972 (4) 8295672; Email: [hhossam@technion.ac.il](mailto:hhossam@technion.ac.il) (H.H.) and [utisch@tx.technion.ac.il](mailto:utisch@tx.technion.ac.il) (U.T.)


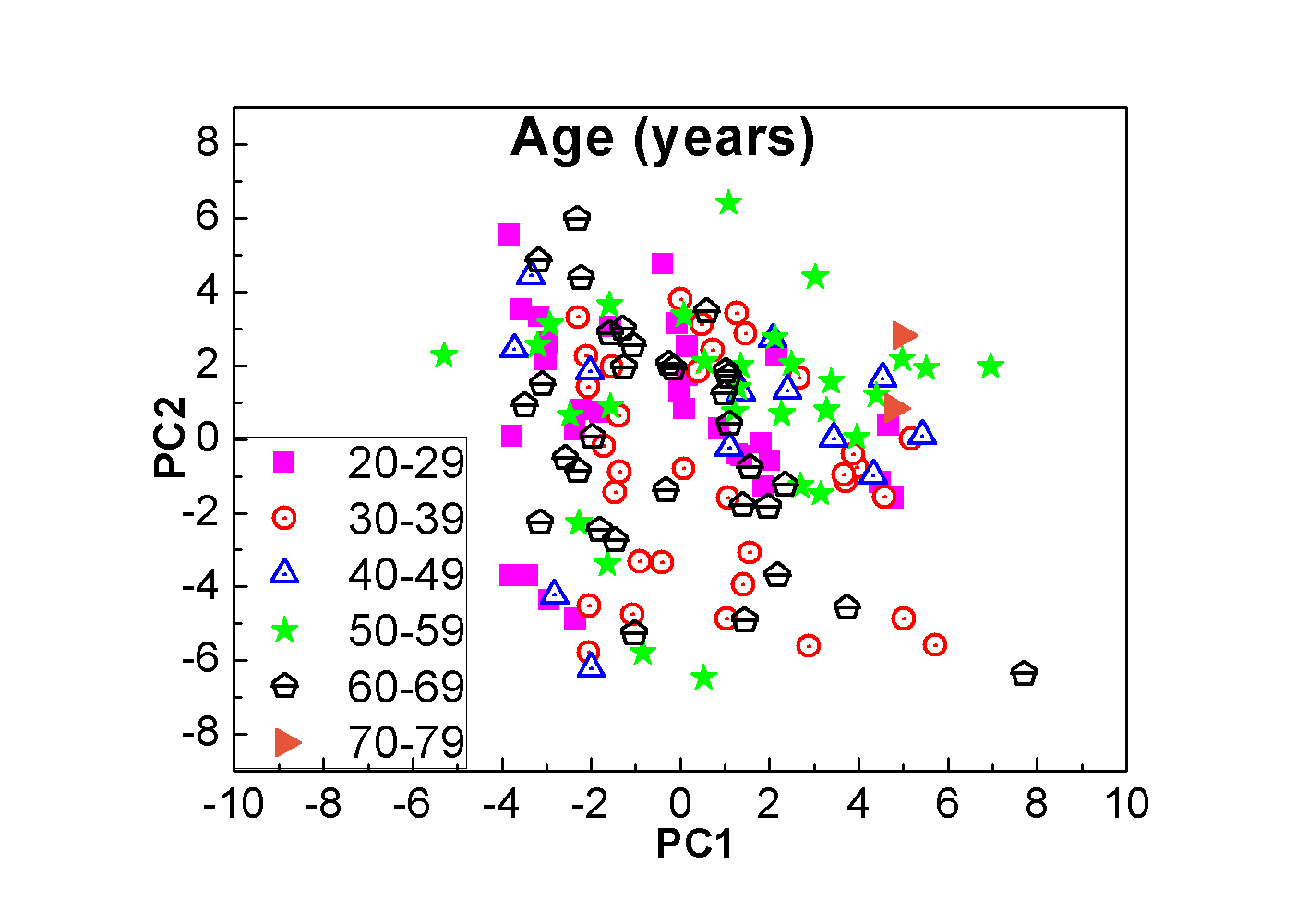

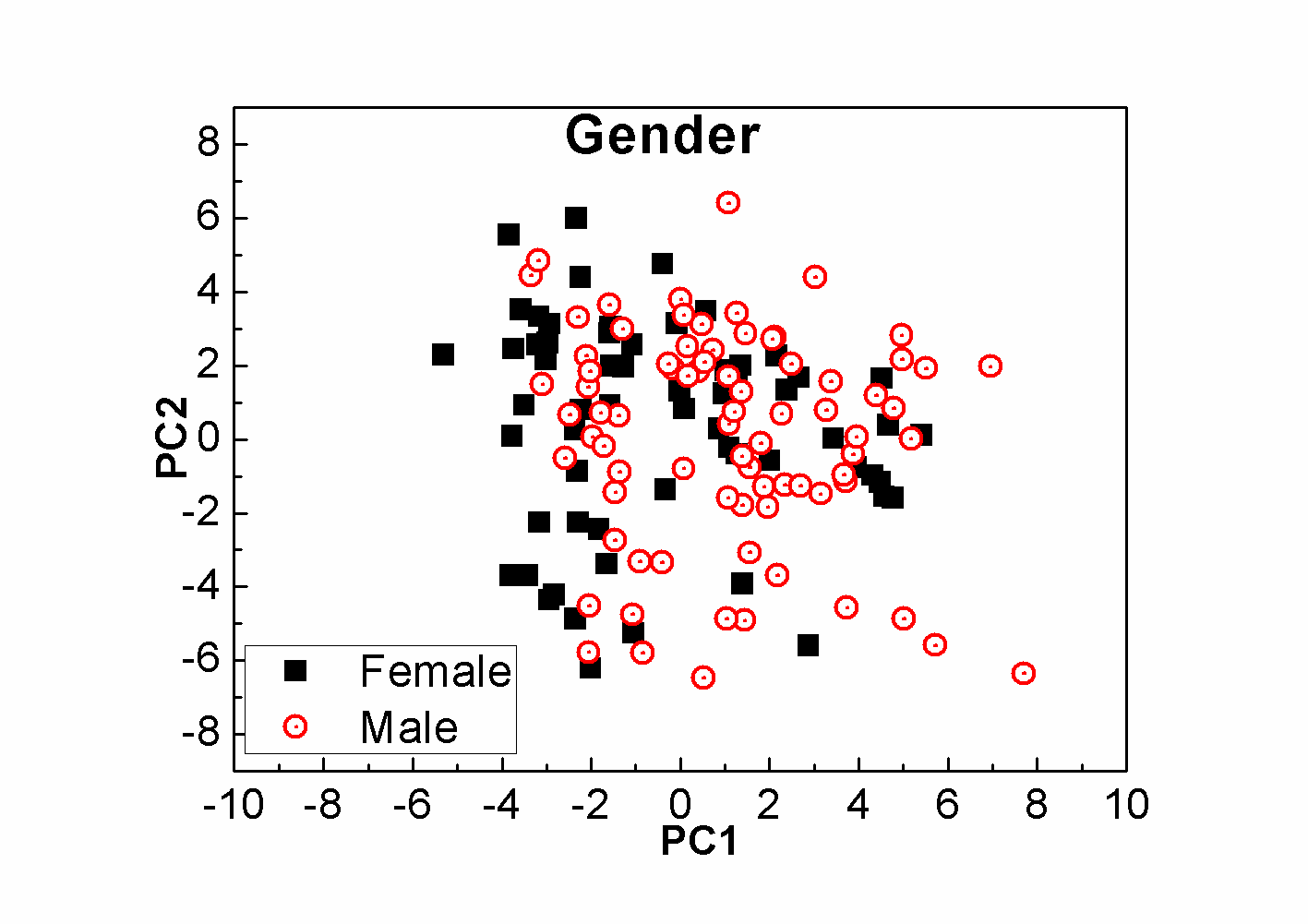

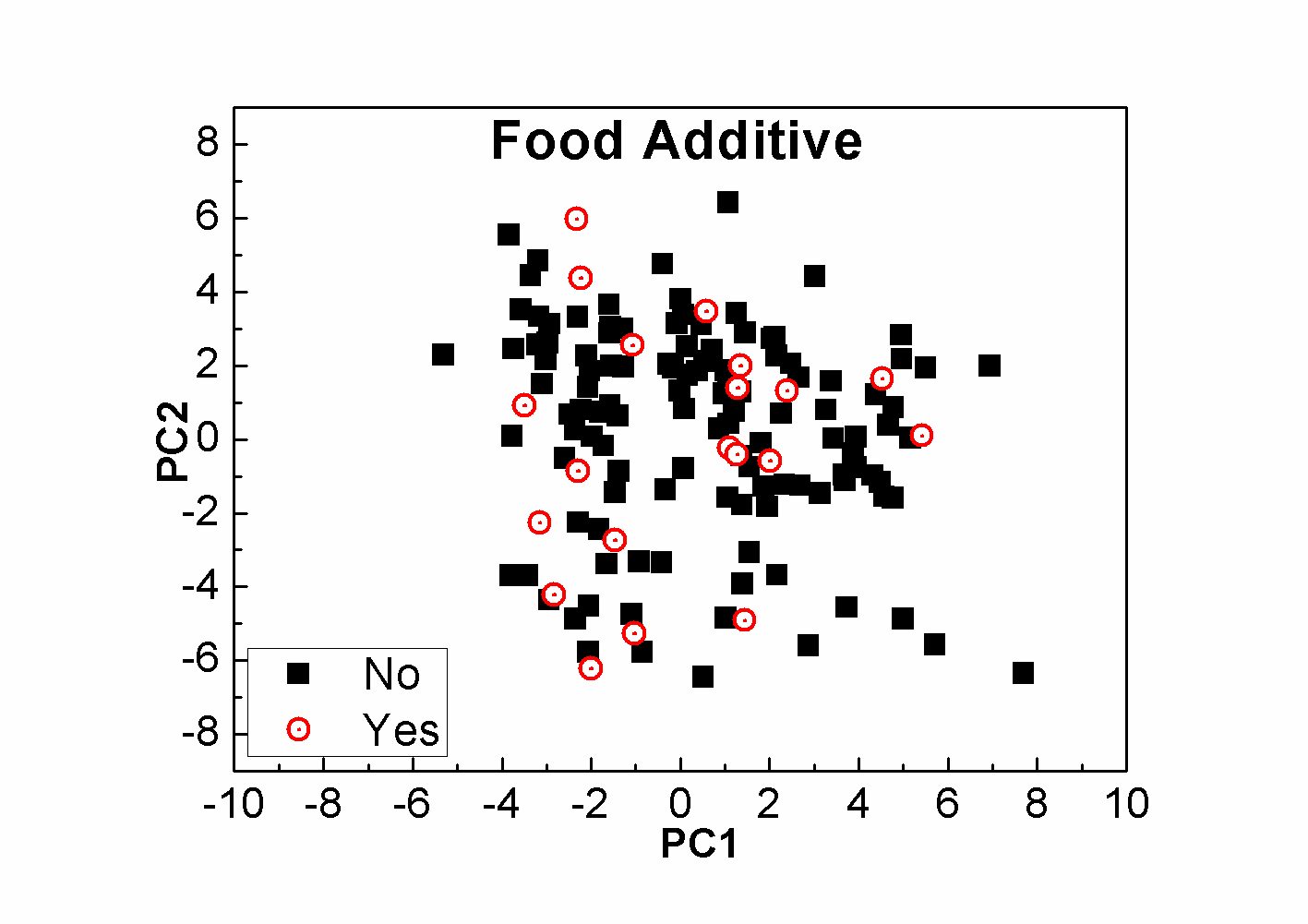

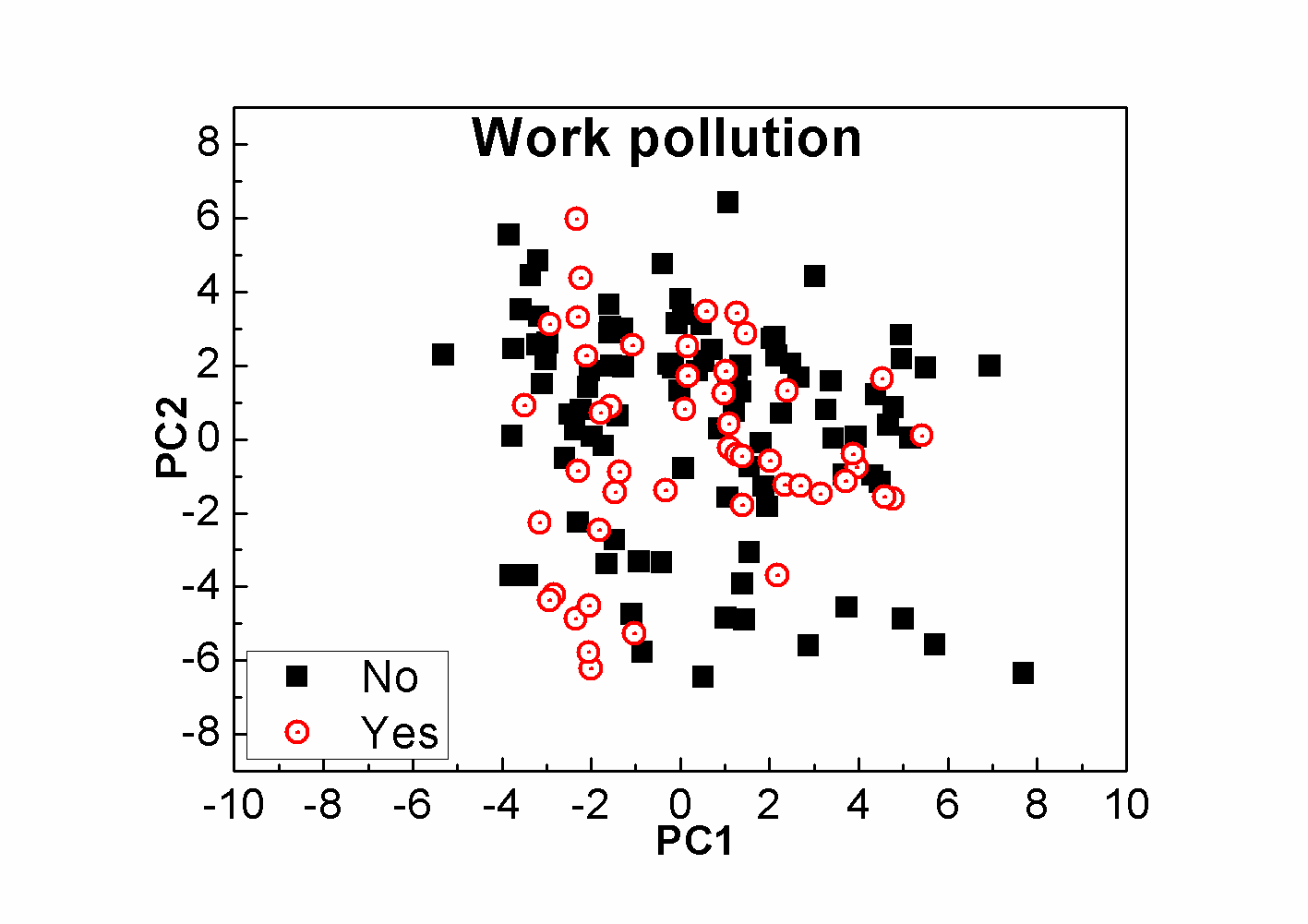

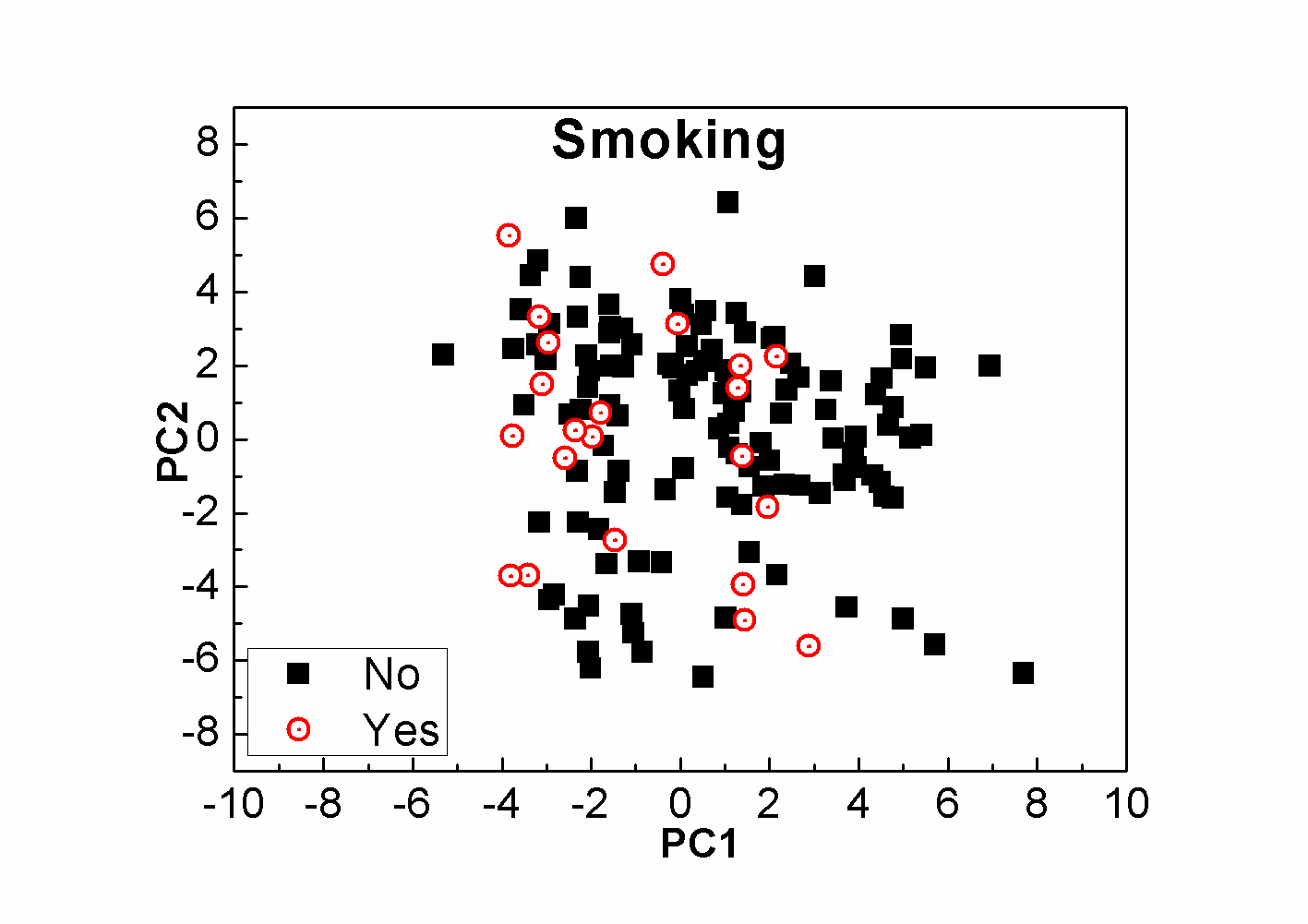


**Figure S1** PCA plots of a representative array of 10 GNP sensors exposed the breath of 59 healthy subjects. Typically, two samples of each subject were analyzed. Plots were analyzed according to different characteristics: age, gender, intake of food additives, work pollution (including long-term exposure to clinical environment), and smoking habits (see Table 2 in Ref. (Pe*ng et* al, 2010)). The first two principal components depicted contained 90% of the total variance in the data. *Partial repoduction of Fig. 4 in Ref. (Peng et al, 2010).*

**Figure S2** PCA plots of the collective response of the NA-NOSE (based on 5 GNP sensors) presented in this study after exposure to the breath of 7 smoking and 19 non-smoking healthy subjects. This NA-NOSE, that is based on 5 GNP sensors clearly identified HNC states (*cf.* Fig. 1 in the article), **but showed no separation** between smokers and non-smokers.


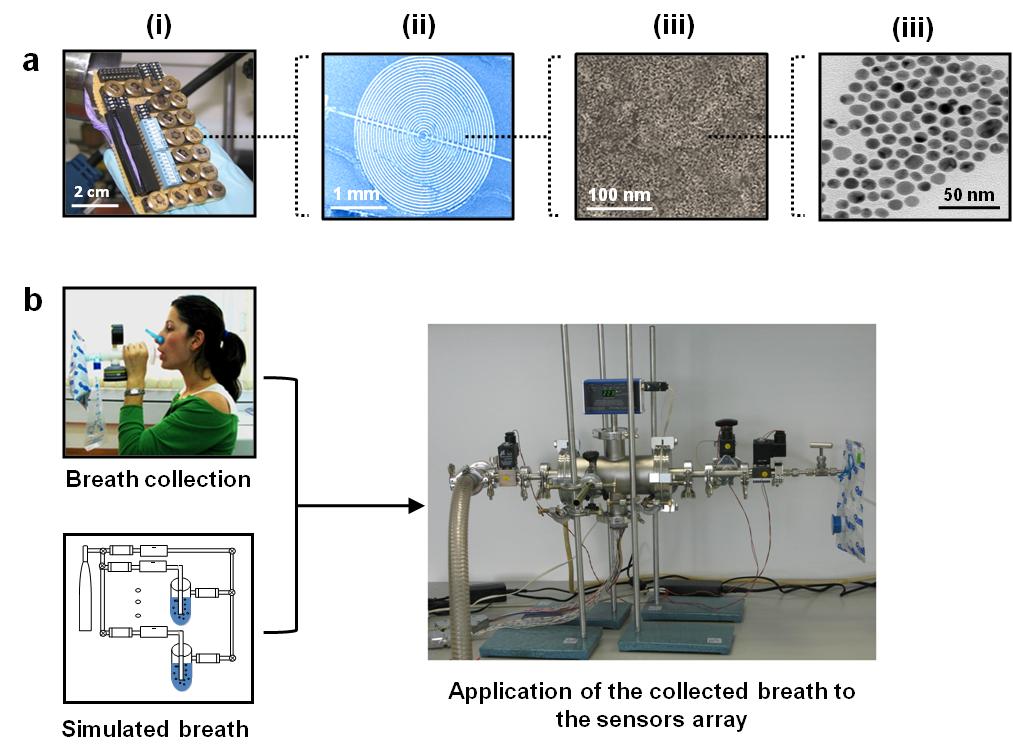


**A**

**B**

**Figure S3** **Illustration of the diagnosis of cancer via breath testing.** **(A)** A photograph of the array of GNP sensors **(i)**, a scanning electron microscopy for a the sensor **(ii)**, a scanning electron microscopy image of a GNP film located between two adjacent electrodes **(iii)**, and a transmission electron micrograph of the monolayer-capped GNPs **(iv)**. NOTE: in the transmission electron microscopy image, the GNPs appear as dark dots while the capping organic molecules appear as a bright medium between the adjacent dark dots. In these films, the metallic particles provide the electric conductivity and the organic film component provides sites for the sorption of analyte (guest) molecules. **(B)** Testing the exhaled breath that was collected from patients, using the array of GNP sensors. *Partial reproduction of Fig. 2 in Ref. (Peng et al, 2009).*

**Figure S4** PCA plots based on the abundances of (**A)** the set of 6 VOCs that differentiate between HNC and healthy controls, (**B**) the set of 7 markers that differentiate between HNC and LC and (**C**) the set of 6 VOCs that was identified in our previous study (Pe*ng et* al, 2010) for LC healthy separation. The PCA analysis was performed on the study group of LC patients (10 smokers and 7 non-smokers), as representative example. The three VOC marker sets that distinguished between (i) HNC and healthy states, (ii) HNC and LC states and (iii) LC and healthy states, **showed no separation** between smokers and non-smokers.

**Table S1** Clinical characteristics of 87 volunteers (22 HNC patients, 25 LC patients and 40 healthy controls), aged 24-78, that were tested for this study.

| Cancer type | GC-MS | NA-NOSE | Gender | Age | Smoker | Ex-smoker | Family cancer history | Sub-site/ Histology | TNM stage* | Overall stage | Medical history and medication |
| --- | --- | --- | --- | --- | --- | --- | --- | --- | --- | --- | --- |
| Head-and-Neck Cancer |  | x | M | 40 | Y | -- | N | Larynx Carcinoma/  SqCC | T4aN2cM0 | IVa |  |
|  | x |  | M | 59 | Y | -- | N | Larynx Carcinoma/  SqCC | T1bn0m0 | I | No medication, no other chronic diseases |
|  |  | x | M | 62 | N | N | N | Oral cavity/SqCC | T4N2M0 | IVa | n/a |
|  | x |  | M | 58 | Y | -- | Y | Oropharyngeal carcinoma/  SqCC | T4aN2cM0 | IVa | No medication, no other chronic diseases |
|  | x |  | M | 72 | Y | -- | N | Hypopharyngeal Carcinoma/  SqCC | T3N0M0 | III | n/a |
|  |  | x | F | 60 | N | N | Y | SqCC | T1 | I | Takes medication for diabetes and cholesterolemia; hyperlipidemia |
|  |  | x | F | 69 | N | Y | Y | Hypopharyngeal Carcinoma/  SqCC | T3N2M0 | IVa | Takes medication for osteoporosis |
|  |  | x | F | 64 | Y | -- | Y | Supraglottis Carcinoma/  SqCC | T3N0M0 | III | No medication, no other chronic diseases |
|  | x |  | M | 68 | N | N | n/a | Maxilla/ Adeno Ca.rcinoma polymorphous type | Recurrent tumor T4 | IVc | Hypertension; Takes Micropirine and vitamins |
|  |  | x | M | 47 | Y | -- | Y | Larynx Carcinoma/  SqCC | T2N0M0 | II | Hypertension; Takes Simvacor, Enaladex, Aspirin, and Micropirin |
|  | x | x | M | 63 | Y | -- | Y | Larynx Carcinoma/  SqCC | T2N0M1 | IVc | No other chronic diseases; No medication |
|  |  | x | M | 57 | N | Y | Y | Larynx Carcinoma/  SqCC | T1N0M0 | I | Takes Cipramil, and Bondormin |
| Cancer type | GC-MS | NA-NOSE | Gender | Age | Smoker | Ex-smoker | Family cancer history | Sub-site/ Histology | TNM stage* | Overall stage | Medical history and medication |
| Head-and-Neck Cancer |  | x | M | 59 | Y | -- | Y | Larynx Carcinoma/  SqCC | T1N0M1 | IVc | Alcohol and drug abuse |
|  |  | x | M | 62 | N | N | N | Oral cavity/SqCC | T4N2M0 | IVa | Hypertension and Hyperlipidemia; Takes Simvacor and Alluril |
|  |  | x | M | 40 | Y | -- | N | Larynx Carcinoma/  SqCC | T4aN2cM0 | IVa | n/a |
|  |  | x | M | 57 | Y | -- | Y | Supraglottis Carcinoma/  SqCC | T3N0M0 | III | No other chronic diseases; No medication |
|  | x | x | M | 63 | Y | -- | N | Larynx Carcinoma/  SqCC | T1N0M0 | IVc | No other chronic diseases; No medication |
|  |  | x | M | 70 | Y | -- | N | Larynx Carcinoma/  SqCC | T1N0M1 | IVc | Hyperlipidemia, alcohol and drug abuse; Takes Simvastatin and calcium supplements |
|  |  | x | M | 70 | N | N | N | Nasophalynx carcinoma/  Sqcc | T2N1M0 | IVa | Hypertension and diabetes; Takes Normiten, Amiodacore and Glucophage |
|  |  | x | M | 59 | Y | -- | Y | Larynx Carcinoma/  SqCC | T1N0M1 | IVc | n/a |
|  | x |  | M | 59 | Y | -- | N | Oral cavity/SqCC | T4N1M0 | Iva | Diabetes; Takes a variety of medications |
|  | x |  | M | 70 | N | N | N | Nasopharynx Carcinoma/  SqCC | T4N2M0 | IVa | Hypertension and Venous Thromboembolism; Takes a variety of medications |
| Lung Cancer | x | x | M | 58 | N | N | Y | NSCLC^§^ | Not relevant | IV | Takes  Normiten, Omepradex, Tevapirin and Simovil |
|  | x | x | M | 64 | Y | -- | N | NSCLC |  | IV | No other chronic diseases; No medication |
| Cancer type | GC-MS | NA-NOSE | Gender | Age | Smoker | Ex-smoker | Family cancer history | Sub-site/ Histology | Not relevant | Overall stage | Medical history and medication |
| Lung Cancer | x | x | M | 71 | N | N | N | NSCLC |  | IV | Takes Coumadin |
|  | x | x | M | 60 | Y | -- | n/a | NSCLC |  | IV | No other chronic diseases; No medication |
|  |  | x | M | 73 | Y | -- | N | NSCLC |  | IV | No other chronic diseases; No medication |
|  |  | x | M | 76 | N | n/a | N | NSCLC |  | IV | Hypertension; Takes Normiten, Osmoadalat, Simovil and Cartia |
|  |  | x | M | 64 | N | n/a | N | SqCC |  | III | COPD\|\|; Takes Analgetics |
|  |  | x | F | 78 | N | n/a | Y | NSCLC |  | III | No other chronic diseases; No medication |
|  | x | x | M | 75 | Y | -- | N | SqCC |  | III | Asthma; Takes Xatral and Simvacor |
|  | x | x | M | 69 | N | n/a | N | NSCLC |  | IV | No other chronic diseases; No medication |
|  | x | x | F | 69 | N | n/a | N | Adeno Carcinoma |  | III | n/a |
|  | x | x | M | 76 | N | n/a | Y | Adeno Carcinoma |  | III | Diabetes; Takes Glucophage |
|  | x | x | M | 74 | Y | -- | N | NSCLC |  | IV | Hypertension; Takes Captopril |
|  | x | x | M | 60 | Y | -- | N | Adeno Carcinoma |  | III | No other chronic diseases; No medication |
|  | x | x | M | 60 | Y | -- | N | SqCC |  | III | No other chronic diseases; No medication |
|  |  | x | M | 58 | Y | -- | N | Adeno Carcinoma |  | III | No other chronic diseases; No medication |
|  |  | x | M | 59 | Y | -- | Y | NSCLC |  | III | No other chronic diseases; No medication |
|  |  | x | M | 59 | Y | -- | N | Adeno Carcinoma |  | III | Hypertension and diabetes; Takes Fusid, Micropirin, Enaladex and Simovil |
|  |  | x | M | 50 | Y | -- | N | SqCC |  | IV | Hypertension and diabetes; Takes Fusid, Micropirin, Enaladex and Simovil |
| Cancer type | GC-MS | NA-NOSE | Gender | Age | Smoker | Ex-smoker | Family cancer history | Sub-site/ Histology | Not relevant | Overall stage | Medical history and medication |
| Lung Cancer | x | x | F | 76 | Y | -- | Y | Adeno Carcinoma |  | IV | No other chronic diseases; No medication |
|  | x |  | M | 57 | Y | -- | Y | Adeno Carcinoma |  | III | Hypertension and hypercholesterolemia; Takes  Simovil and Aspirin |
|  | x |  | M | 70 | Y | -- | n/a | NSCLC |  | n/a | n/a |
|  | x |  | M | 71 | N | N | N | Adeno Carcinoma |  | IV | n/a |
|  | x |  | M | 61 | Y | -- | n/a |  |  |  | No other chronic diseases; No medication |
|  | x |  | M | 61 | N | Y | N | SqCC |  | IV | Hypertension and hyperlipidemia; Takes Normiten, Omnic and Simovil |
| Healthy Controls |  | x | F | 29 | N | n/a | N | Not relevant | | | Asthma; Takes Simvicourt |
|  |  | x | M | 38 | N | n/a | N |  |  |  | No chronic diseases; No medication |
|  |  | x | M | 34 | N | n/a | N |  |  |  | No chronic diseases; No medication |
|  |  | x | F | 46 | N | n/a | N |  |  |  | No chronic diseases; No medication |
|  |  | x | F | 37 | N | n/a | N |  |  |  | No chronic diseases; No medication |
|  |  | x | M | 37 | Y | -- | N |  |  |  | No chronic diseases; No medication |
|  |  | x | F | 38 | N | n/a | N |  |  |  | Asthma and sinusitis; Takes Mizollene, Seretide Diskus |
|  |  | x | M | 31 | Y | -- | N |  |  |  | No chronic diseases; No medication |
|  |  | x | M | 25 | Y | -- | N |  |  |  | Takes Omeprazole |
|  |  | x | F | 24 | N | n/a | N |  |  |  | No chronic diseases; No medication |
|  |  | x | F | 26 | N | n/a | N |  |  |  | No chronic diseases; No medication |

| Cancer type | GC-MS | NA-NOSE | Gender | Age | Smoker | Ex-smoker | Family cancer history | Not relevant | Medical history and medication |
| --- | --- | --- | --- | --- | --- | --- | --- | --- | --- |
| Healthy Controls |  | X | M | 24 | N | n/a | N |  | No chronic diseases; No medication |
|  |  | X | F | 47 | N | n/a | N |  | Hypothyroidism; Takes Altroxen |
|  |  | X | F | 40 | Y | -- | Y |  | No chronic diseases; No medication |
|  |  | X | F | 50 | Y | -- | N |  | No chronic diseases; No medication |
|  |  | x | M | 33 | N | n/a | N |  | No chronic diseases; No medication |
|  |  | x | F | 67 | Y | -- | N |  | Takes cholesterole regulation medication |
|  |  | x | M | 54 | N | n/a | N |  | No chronic diseases; No medication |
|  |  | x | F | 63 | N | n/a | Y |  | Hypertension, had breast cancer 28 years ago; Takes Osmo Adalat, Simovil |
|  |  | x | F | 29 | N | n/a | Y |  | No chronic diseases; No medication |
|  |  | x | M | 38 | Y | -- | Y |  | Hypertension ; Takes Oxar |
|  |  | x | M | 45 | N | n/a | N |  | No chronic diseases; No medication |
|  |  | x | M | 38 | N | n/a | Y |  | No chronic diseases; No medication |
|  |  | x | F | 61 | N | n/a | Y |  | Takes hormonem preparates, Caltrate, Vitamin D and Fosolan |
|  | x |  | F | 53 | N | -- | N |  | No other chronic diseases; No medication |
|  | x | x | F | 46 | N | n/a | Y |  | No chronic diseases; No medication |
|  | x |  | M | 53 | N | -- | N |  | No other chronic diseases; No medication |
|  | x |  | F | 50 | N | n/a | N |  | Takes Altroxen |
|  | x |  | M | 55 | N | -- | N |  | No other chronic diseases; No medication |
|  | x |  | M | 58 | N | -- | Y |  | No other chronic diseases; No medication |
|  | x |  | F | 49 | N | n/a | N |  | No chronic diseases; No medication |

| Cancer type | GC-MS | NA-NOSE | Gender | Age | Smoker | Ex-smoker | Family cancer history | Not relevant | Medical history and medication |
| --- | --- | --- | --- | --- | --- | --- | --- | --- | --- |
| Healthy Controls | x |  | M | 50 | N | n/a | Y |  | No chronic diseases; No medication |
|  | x |  | F | 52 | N | n/a | Y |  | Hypothyroidism ; Takes Latroxin |
|  | x |  | F | 61 | N | n/a | N |  | Diabetes |
|  | x |  | F | 59 | N | n/a | N |  | No chronic diseases; No medication |
|  | x |  | F | 36 | N | n/a | N |  | No chronic diseases; No medication |
|  | x |  | F | 39 | N | n/a | N |  | No chronic diseases; No medication |
|  | x |  | M | 63 | N | n/a | Y |  | Hypertension; Takes blood pressure regulating medications |
|  | x |  | F | 47 | N | n/a | Y |  | Takes Cordil; heart condition |
|  |  | x | M | 67 | N | n/a | N |  | No chronic diseases; No medication |

* HNC is usually staged according to the TNM system, whereby T stands for [tumor](http://en.wikipedia.org/wiki/Tumor) size, N for regional lyph node involvement and M for distant metastasis spread (Rid*ge et* al, 2009).

^‡^ SqCC = Squamous cell carcinoma

^§^ NSCLC = [non-small cell lung carcinoma](http://en.wikipedia.org/wiki/Non-small_cell_lung_carcinoma)

^||^ COPD = Chronic obstructive pulmonary disease

**Table S2** Tentative identification of smoking related compounds in exhaled breath, using GC-MS/SPME (for experimental details see section 2.5 in the article). Preliminary results from a pilot study using eight healthy smokers and eight healthy non-smoking controls that have never smoked in their lives. This study is on-going and the results for an extended study population will be published elsewhere. Note, that the pre-concentration (i. e. the type of SPME fiber) used in this study differs from the pre-concentration used by other research groups (*see* for example Refs. (Ama*nn et* al, 2010; Fuc*hs et* al, 2010; Kischk*el et* al, 2010) and references therein). This may explain the identification of different markers of smoking.

| Tentative compound name | Main mass (m/z) | Occurrence |
| --- | --- | --- |
| 2,3,5,8-Tetramethyl-decane | 71 | Found in the breath of > 80% of smokers and < 20% of non-smokers |
| 6,10-Dimethyl-4-undecanol | 43 |
| N-Butyl-benzenesulfonamide, | 77 | Found in the breath of > 80% of smokers and >80% of non-smokers and shows significant differences in abundance (p = 0.023) |
| 1-Acetyl-4-(4-propylcyclohexyl)- benzene | 229 | Found in the breath of > 80% of smokers and >80% of non-smokers and shows sub-significant differences in the averages of abundances > (0.1 > p >0.05) |
| 2-Methyl-pentane | 43 |
| 2,3-Dihydro-1,1,3-trimethyl-3-phenyl-1H-indene | 3 |
| Diisopropylhydroxy-phosphine | 18 |
| 2,2-Dimethylcyclobutyl-methylamine | 73 |

**REFERENCES**

Amann A, Miekisch W, Pleil J, Risby T, Schubert J (2010) Chapter 7: Methodological issues of sample collection and analysis of exhaled breath *European Respiratory Society Monograph* 49: 96-114

Fuchs P, Loeseken C, Schubert JK, Miekisch W (2010) Breath gas aldehydes as biomarkers of lung cancer. *Internat J Cancer* 126: 2663-2670

Kischkel S, Miekisch W, Sawacki A, Straker EM, Trefz P, Amann A, Schubert JK (2010) Breath biomarkers for lung cancer detection and assessment of smoking related effects - confounding variables, influence of normalization and statistical algorithms. *Clin Chim Acta* 411: 1637-1644

Peng G, Hakim M, Broza YY, Billan S, Abdah-Bortnyak R, Kuten A, Tisch U, Haick H (2010) Detection of lung, breast, colorectal, and prostate cancers from exhaled breath using a single array of nanosensors. *British Journal of Cancer* 103: 542 – 551

Peng G, Tisch U, Adams U, Hakim M, Shehada N, Broza YY, Billan S, Abdah-Bortnyak R, Kuten A, Haick H (2009) Diagnosing lung cancer in exhaled breath using gold nanoparticles. *Nature Nanotechnol* 4: 669-673

Ridge JA, Glisson BS, Lango MN, Feigenberg S (2009) Head and neck tumors. In *Cancer Management: A Multidisciplinary Approach Medical, Surgical & Radiation Oncology*, Pazdur R, Wagman LD, Camphausen KA, Hoskins WJ (eds), 12 edn.: CMP Healthcare Media LLC
